# Radon Natural Radioactivity Measurements for Evaluation of Primary Pollutants

**DOI:** 10.1155/2013/626989

**Published:** 2013-07-11

**Authors:** Fenjuan Wang, Zhenyi Zhang, Maria Pia Ancora, Xiaodong Deng, Hua Zhang

**Affiliations:** ^1^National Climate Center, China Meteorological Administration, Beijing 100081, China; ^2^Tsinghua University, Beijing 100084, China; ^3^Lanzhou Environment Monitoring Centre, Lanzhou Gansu 730000, China

## Abstract

Radon is naturally released from the soil into the surface layer of the atmosphere, and by monitoring the natural radioactivity data of radon and its shot-live decay products we can get valuable information about the dilution properties of the lower boundary layer. This paper explores the dispersion characteristics of the lower layer of the atmosphere in Lanzhou, China, and the close relationship with the patterns of primary pollutants' concentrations. Measurements were conducted from July 2007 to May 2008 at one station and a fifty-day campaign was carried out at two stations in Lanzhou. The interpretation of radon radioactivity measurement showed that the measured atmospheric stability index (ASI) data at two stations in Lanzhou had statistically significant correlation, and well described the lower atmospheric layer mixing property in the area. The temporal trend of PM_10_ data was consistent with the temporal trend of ASI, with almost twice as high values in December than it in August. The results show that the ASI allows to highlight the dilution factor playing an important role in determining primary pollution events, and the mixing properties of the lower boundary layer is the key factor determining PM_10_ concentration in urban areas.

## 1. Introduction

China is a unique platform to conduct air pollution studies, and there are many air pollution scientific issues such as primary particles and secondary organic aerosol formation in Chinese mega cities that still remain to be deeply explored [1, 2]. Lanzhou is one of the most polluted cities in China, which is heavily burdened with emissions from big chemical plants and characterized by some of the worst dispersion conditions in China. The long term PM_10_ concentration trend in Lanzhou between 2001 and 2007 decreased, but it still has 25% days exceeding the national Grade II air quality standard [3]. Several studies have identified the main particle sources and strategies for reducing particles [4, 5], but less effort has been made to understand the main factors determining particle variations in Lanzhou and other polluted cities in China, which are urgently in need of pollution control strategies.

In this paper we present a technique to study the dispersion properties of the boundary layer based on the monitoring of natural radioactivity due to short-lived decay products of radon. Radon gas, which is produced in the soil by radioactive decay of ^226^Ra, a member of the ^238^U series, is released from the soil into the surface layer of the atmosphere [6]. There are factors such as soil composition, moisture content, porosity and temperature and so on that affects the radon emission rate. For a given geographical location and for weeks of observation, however, the emission flux of radon can be considered to be constant and the air concentration of radon and ^222^radon short-lived daughters (^218^Po, ^214^Pb, ^214^Bi and ^214^Po) can be assumed to depend only on the dilution factor [7]. The dilution properties of the lower atmosphere can, therefore, be characterized by monitoring natural radioactivity due to radon progeny absorbed to atmospheric particles [1, 2, 8], and the dilution properties can be used to analyze primary pollutants pollution [9, 10].

This paper reports the results of a research project aimed to explore the reasons of heavy air pollution in the city of Lanzhou, China, by studying the mixing properties of the lower boundary layer by monitoring the air concentration of radon progeny between July 2007 and May 2008. The index allows us to uncouple the emission factor and the dilution factor, which determines the atmospheric concentration of primary pollutants in the lower atmospheric layer. 

## 2. Methods

### 2.1. Radon Dilution Method

The mass of pollutants in the atmosphere is influenced by emission fluxes, physical-chemical transportation, and deposition processes, while the volume of pollutants is influenced by advection (mainly horizontal movements of the air masses, mechanical turbulence due to winds), and convection (mainly thermal turbulence, vertical movements due to the heating of the lower air masses). The concentration of pollutants can be described by the following (Perrino et al. [11]):

(1) $$\begin{array}{c} \frac{\partial C_{i}}{\partial t}=\alpha \left[\phi_{i}\left(t\right)\right]-\beta \left\{C_{i}\right\}+Adv+\sum F_{i}-\sum R_{i}-D_{s}, \end{array}$$

where ∂*C*_*i*_/∂*t* is the instantaneous pollutants' concentration; *ϕ*_*i*_(*t*), ∑*F*_*i*_, ∑*R*_*i*_, and *D*_*s*_ are emission fluxes from the sources, formation processes, chemical removal processes, and surface deposition loss, respectively. *Adv*, *β*{*C*_*i*_}, and *α* are transport process parameters, mixing process parameters, and surface layer stability parameters, respectively. For primary pollutants, they are mostly inactive and their chemical processes are very slow; then ∑*F*_*i*_, ∑*R*_*i*_, and *D*_*s*_ can be neglected; thus, the concentration change in the atmosphere can be described by

(2) $$\begin{array}{c} \frac{\partial C_{i}}{\partial t}=\alpha \left[\phi_{i}\left(t\right)\right]-\beta \left\{C_{i}\right\}+Adv_{s}. \end{array}$$

As said, the emission rate of radon varies in different places, depending on the factor such as soil composition, soil moisture content, and porosity, but the variations can be considered to be negligible in a certain area on the scale of several kilometers and the scale of weeks [12]. Thus the concentration of radon in atmosphere is mainly determined by the dilution factor mixing of the boundary layer, and radon can be considered a good natural tracer of the mixing properties of the lower boundary layer.

### 2.2. Experimental

**Figure 1:**
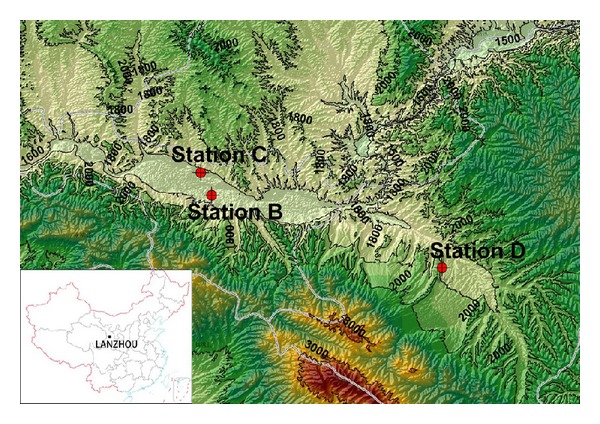
Location of measurement stations in the contour map of Lanzhou (the inset map shows the location of Lanzhou in China).

Lanzhou, located in the north-western part of China (Figure 1), the capital city of Gansu province, has a total area of 13,100 km^2^ and an urban population of 2.01 million. Yellow River flows through Lanzhou eastward in a narrow flat valley with highly rugged mountain ranges to the south and north. The City occupies 2 basins in the east and west forming a shape of a dumbbell, stretching 35 km from east to west and 8 km from north to south. There are some other tributaries that drain the municipality leaving abundant water resources and hydrological power to harness. Lanzhou has a semiarid continental monsoon climate. The annual precipitation is 230–350 mm and contrasts with significant evaporation of about 1400 mm per year. Sixty percent of the precipitation falls in July, August, and September. The annual average temperature in the middle river valley area is between 8°C and 9°C. Petroleum, chemical industry, mechanics, and metallurgy are four key industries. Other industries include textiles, food, medicine, electricity, cement, steel and iron, nonferrous metal, coal, and building materials. Some of these industries emit great amounts of SO_2_,   NO_*x*_, and PM_10_, and other suspended particles and gaseous pollutants. Large tracts of bare land within and around Lanzhou also contribute particles to the atmosphere.

Under the Sino-Italian Cooperation Program on Environmental Protection, an air quality monitoring network was established downstream of a preliminary assessment structure which was carried out according to Framework Directive 96/62/EC [13]. Three types of fixed monitoring stations were designed and built (Figure 1). Station B and station C are located in the eastern part of the city: station B, representative of residential population exposure, is hosted in the flower nursery of a residential park in Anning district, and station C, representative of traffic pollution exposure is located in Qilihe district on the side of one of the main arteries of the city, Yicheng road. Station D, representative of rural areas/regional background pollution is located in Yuzhoug countryside, west of the Lanzhou central urban area, and is hosted in Lanzhou University, near the meteorological department field observation equipment.

Measurements were carried out from July 11, 2007 to May 31, 2008 in station C and from July 11 to September 3, 2007 at station B and station C simultaneously. The atmospheric stability index (ASI) was measured by an atmospheric stability monitor (SM200, OPSIS, Sweden). PM_10_ was measured using a continuous Rupprecht and Patashnick TEOM particle monitor (TEOM 1400a, USA) at station D. Instruments were calibrated at the laboratory and maintained regularly. SYSTAT 12 (Cranes Software International Ltd.) was used for statistic analysis in this study. 

## 3. Results and Discussions

### 3.1. Temporal Variation of ASI Measurement

**Figure 2:**
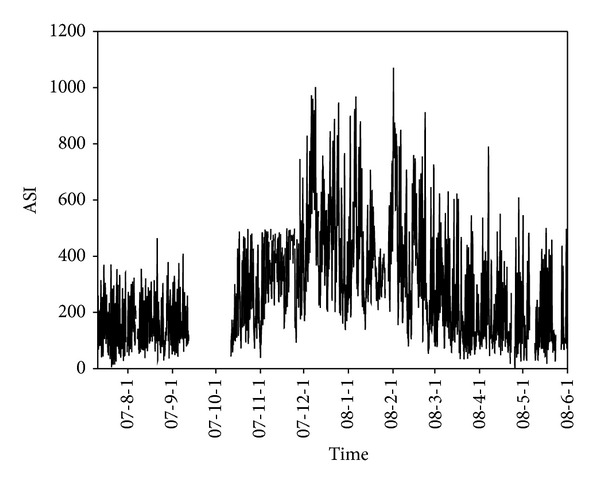
ASI measurement at station C (July 11, 2007, to May 31, 2008, data missing from September 12 to October 12, 2007, for instrument maintenance or error).

**Figure 3:**
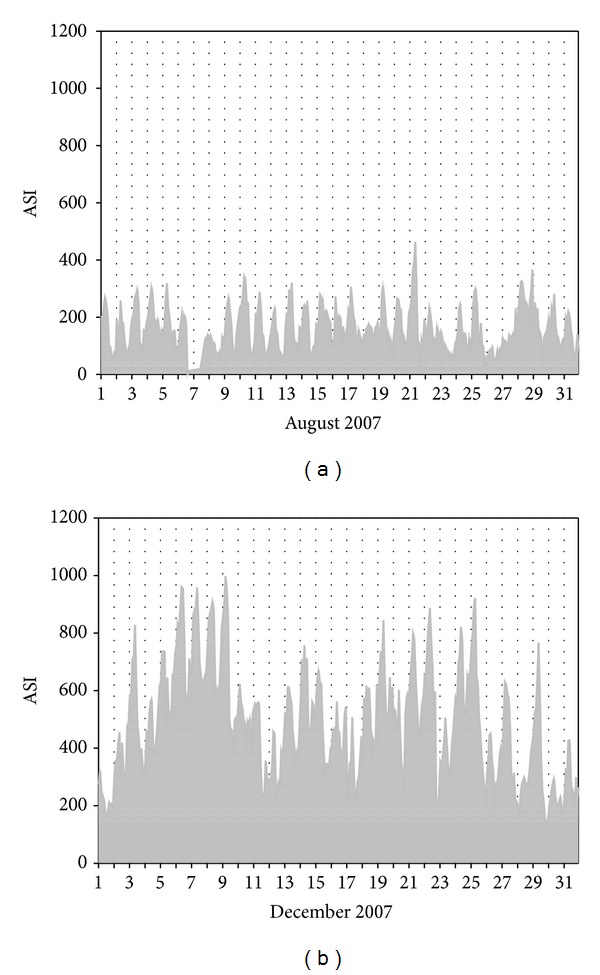
Temporal trend of ASI in August and December, 2007, (data missing on August 6-7 for maintenance of the instrument).

The temporal pattern of ASI measurement from July 11, 2007 to May 31, 2008 is shown in Figure 2. The ASI values show clear seasonal variation, minimum in July and August and maxima in December and January then decreased again. The atmospheric mixing is strongly dependent on the duration and strength of sunlight (convective mixing), which might cause the differences between warm months and cool months. The temporal trends of natural radioactivity during the months of August and December are compared in Figure 3. During August and during all warm months, natural radioactivity shows a similar modulated diurnal trend and most days are very similar. During day time the convective mixing of the lower atmosphere is strong and minimum values are recorded; during night time the atmosphere is relatively stable and maximum values are recorded. The mixing period starts very early in the morning and lasts until the late evening consistent with the long sunshine time during August. During December the measured ASI values almost doubled compared to August. Lanzhou is located in a mountain valley (Figure 1), and the inverse layer is very low in winter time. Atmosphere is stable in most of the time, and during the day hours natural radioactivity exhibits high values (e.g., December 6, 7, 8, 9). In December, the diurnal mixing generally occurs during a few hours during the day and also weak, which is reflected by the very narrow window of ASI values during daytime.

### 3.2. Spatial Variation of ASI Measurement

**Figure 4:**
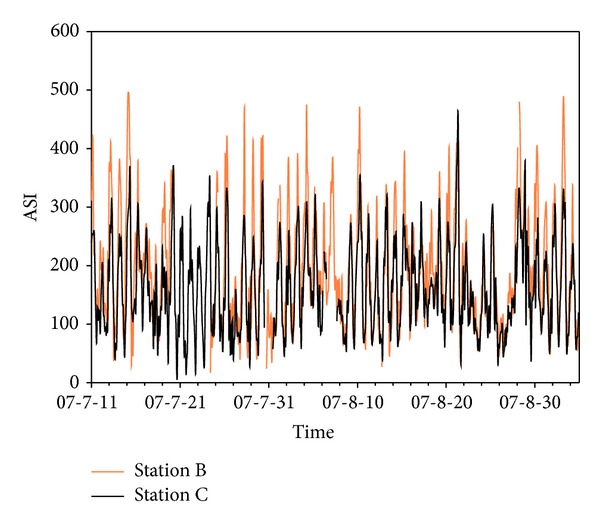
ASI measurements at station B and station C (July 11 to September 3, 2007).

**Figure 5:**
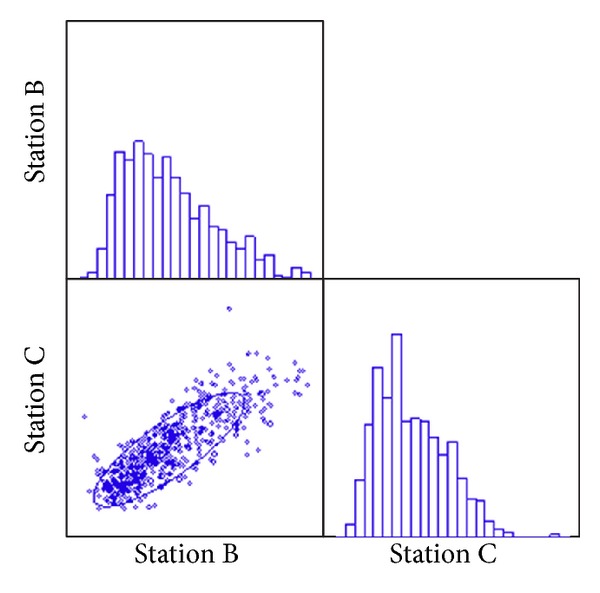
Pearson correlation matrix of ASI measurement data at station B and station C (July 11 to September 3, 2007).

A measurement campaign of ASI was carried out during July 11 to September 3, 2007, at station B and station C simultaneously with same type of instruments. The result shows that ASI values had a similar time trend and diurnal variation at both stations (Figure 4). Daily maxima were generally recorded higher at station B than station C, which might be due to the micropositioning of the stations. Station C is located right by a major road artery of the city and, therefore, is more likely influenced by advection mixing. The data distributions were similar at station B and station C (Figure 5). OLS regression test (multiple *R* = 0.83, *P* = 0.001) indicates that the correlation of the measurements at the two stations were statistically significant. This comparison releases the evidence that the measurement in station C is representative for Lanzhou area and being a valuable index for low layer atmospheric mixing property in Lanzhou area.

### 3.3. ASI Measurement Correlation with PM_10_ Concentration

**Figure 6:**
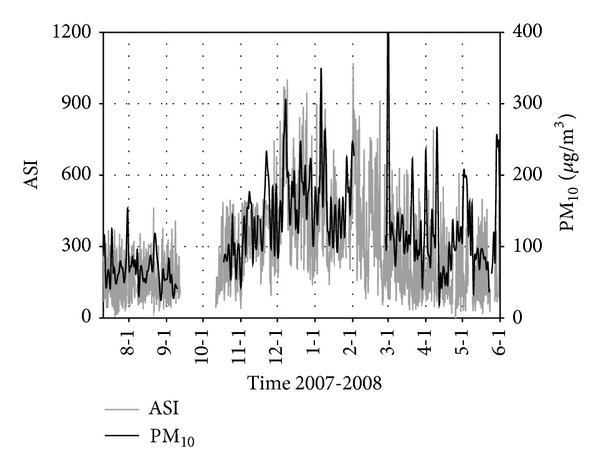
PM_10_ and ASI measurement from July 11, 2007, to May 31, 2008 (data missing for instrument maintenance).

The comparison between the radon radioactivity and the average PM_10_ concentration during the whole period from July 2007 to May 2008 is shown in Figure 6. The correlation between the atmospheric stability index (ASI) and the PM_10_ concentration is statistically significant. In addition Pearson correlation analysis shows that the daily average of ASI and PM_10_ values gained positive correlation coefficients (0.64) and *P* values below 0.01, indicating that ASI and PM_10_ tend to increase and decrease together. 

**Figure 7:**
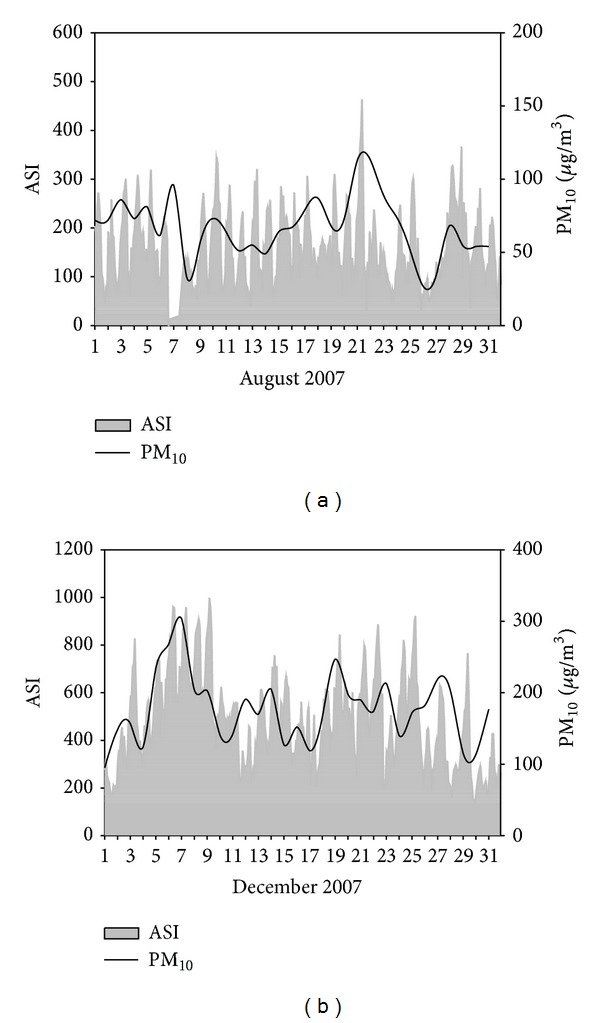
PM_10_ and ASI (Lanzhou, China, August and December, 2007, data missing on August 6-7 for maintenance of the instrument).

During winter months, daily average concentration of PM_10_ reaches 180 *μ*g/m^3^ in December, and during summer months, PM_10_ daily average concentration is as low as 68 *μ*g/m^3^ in August. PM_10_ daily average in December is 2.6 times greater than in August, which is consistence with the ratios of daily ASI values, which in December are 3 times those measured in August (Figure 7). In summer time, the solar radioactivity is high and inversion is short, and thus, the atmosphere is relatively unstable. While in winter time, the average wind speed is 0.98 m/s and the inversion layer is low around 700 m and forms frequently thus the atmosphere is very stable and unfavorable to pollutants' dispersion in the mountain valley [14]. The special topographic conditions of Lanzhou make the dispersion of these atmospheric pollutants into the free atmosphere difficult due to the formation of a stable and strong inverse thermal layer in the valley. This situation is particularly exacerbated in winter when there is great demand for heating. The different duration of the atmospheric mixing phase over a year has an important consequence for atmospheric primary pollutant's concentration. On some individual days, PM_10_ concentration also traces the ASI trend; for example, on August 21 PM_10_ reached a maximum at 113 *μ*g/m^3^ when ASI peaked in whole August, and PM_10_ concentration peaked on December 7 when ASI reached the maximum after days of increasing. There were also some exceptions; for example, on February 29 PM_10_ concentration recorded the highest without an increase of ASI, which due to the dust transportation from Xinjiang dust area in north-west of Lanzhou as the 24-hour back trajectory traced (http://ready.arl.noaa.gov/HYSPLIT_traj.php). The different trend of radon measurement and particle matter concentration was also used to study regional pollution transport in by Manigrasso et al. [10]. The close correlation between PM_10_ and ASI measurement in this study showed that the mixing of the lower atmospheric layer is the major factor in determining the average concentration of nonreactive primary pollutants in Lanzhou. 

## 4. Conclusions

This paper presents the measurement of atmospheric stability by natural radioactivity data of radon from July 11, 2007, to May 31, 2008, in one of the most polluted cities in north-central China, Lanzhou. The atmospheric stability index was used to analyze the primary pollutants PM_10_. The results release the different duration of the atmospheric mixing phase over a year which has an important consequence for atmospheric primary pollutant's concentration. PM_10_ daily average in December is 2.6 times that of August, which is consistent with the ratios of ASI daily values, 3 times higher in December compared to August. The correlation between PM_10_ and ASI measurement shows that dispersion condition in the lower atmospheric layer is the main parameter in determining the average concentration of nonreactive primary pollutants in Lanzhou. The analysis presented in this paper provides evidence that radon measurement can be a valuable tool to interpret, forecast pollution phenomena, as it is a reliable proxy for low boundary layer atmospheric mixing property in the Lanzhou area. We can conclude that the clear difference in seasonal trends of the atmospheric mixing properties, therefore, closely related primary pollutants concentrations, can and should be taken into consideration in tailoring policies and strategies to reduce air pollution phenomena in the city through appropriate urban (location of major emitting sources) and industrial planning (seasonal shifts).
